# The Shifting Core: Antigenic Variability of the Influenza Virus Nucleoprotein Despite Evolutionary Conservation

**DOI:** 10.3390/antib15030041

**Published:** 2026-05-15

**Authors:** Alexandra Rak, Veronika Muzurova, Svetlana Donina, Polina Prokopenko, Irina Isakova-Sivak, Larisa Rudenko

**Affiliations:** Institute of Experimental Medicine, 12 Acad. Pavlov Street, Saint-Petersburg 197022, Russia; nik.fox.m@yandex.ru (V.M.); isakova.sivak@iemspb.ru (I.I.-S.);

**Keywords:** influenza virus, nucleoprotein, immunogenicity, humoral immunity, cross-reactivity, antiviral antibodies

## Abstract

Background. The highly mutable influenza virus causes severe annual infections worldwide and results in substantial socioeconomic losses. The spread of infection could be effectively controlled by cross-protective vaccines and universal diagnostic test systems based on the nucleoprotein (NP) as one of the most conserved viral antigens. However, NP also undergoes slow evolutionary changes, and little is known about the influence of these mutations on its antigenicity and immunogenicity. Methods. We expressed the full-length recombinant 6xHis-tagged NPs of ten evolutionary distant influenza A strains of different subtypes in *E. coli* BL21(DE3) cells and purified these proteins by immobilized metal affinity chromatography. The obtained antigens were identified by mass spectrometry and serological methods. NPs served as antigens for three immunizations of BALB/c mice (15 µg/animal at 14-day interval) and as capturing proteins in ELISA at 2 µg/mL, in order to study the effect of adaptive mutations on the antigenic and immunogenic properties of NPs. Results. A pronounced cross-reactivity of anti-NP antibodies induced in mice by immunization with different NPs was revealed. At the same time, we observed the differences in the humoral immunogenicity of NP, which are in line with the accumulation of evolutionarily driven NP mutations. In general, antibody affinity to heterologous NPs was reduced, indicating the differences in the specificity of anti-NP immunoglobulins, which may be caused by evolutionarily determined variability of immunogenic epitopes leading to the emergence of escape mutations. Conclusions. Overall, our results reflect the slightly evolving nature of the NP antigen, which influences the specificity spectrum of anti-NP antibodies and should be considered as a limitation for the development of NP-based cross-protective vaccines and test systems.

## 1. Introduction

The highly mutable influenza virus causes severe infections annually worldwide and results in significant damage to global economies [1,2]. The spread of infection may be effectively controlled by the cross-protective vaccines and universal diagnostic tools based on the nucleoprotein (NP) as one of the most conserved and immunogenic viral antigens [3]. This viral component is known to cause active production of specific T-effectors and antibodies upon natural influenza infection and vaccination; although the latter lack neutralizing activity, they can mediate innate immune responses such as complement-dependent cytotoxicity or antibody-dependent cellular cytotoxicity/phagocytosis [4,5,6]. Despite its unexposed nature, NP also undergoes slow evolutionary changes [7], and little is known about the influence of these adaptive mutations on its antigenicity and immunogenicity.

The strains that serve as the basis for seasonal whole-virion influenza vaccines are products of the genetic reassortment of circulating viral variants and vaccine donors. Traditionally, their genome consists of the segments encoding surface proteins (hemagglutinin and neuraminidase) of actual epidemic influenza viruses and genes of non-structural and internal proteins (in particular, NP antigen) inherited from the vaccine donor [8]. The most popular donor strains for influenza vaccines worldwide are the viruses isolated in 1933–1960, namely A/WSN/1933 (H1N1) (used to produce modern nanoparticle-based vaccines [9]), A/PR/8/34 (H1N1) (a highly reproductive donor for the production of IIVs [10]), and A/Leningrad/134/17/57 (H2N2), licensed for the LAIV production [11], as well as the attenuation donor for the American LAIV A/Ann Arbor/6/60 (H2N2) [12]. The donor NP composition may have significantly changed from that of circulating viruses for more than 60 years. If these NP substitutions affect immunogenic T- or B-cell epitopes, differences in the composition of “donor” and “epidemic” NP antigens may reduce the efficacy of immune stimulation by vaccines containing antigenically outdated nucleocapsid protein [13]. This raises the question of whether NP should be updated in whole-virion influenza vaccine formulations.

To test the hypothesis about the dissimilarity of humoral responses caused by NP of different influenza A subtypes and the influence of evolutionary variability on its antigenic properties, we analyzed the humoral immunogenicity of recombinant NP from different influenza A strains and the cross-reactivity of anti-NP antibodies induced by these antigens in mice.

## 2. Materials and Methods

### 2.1. NP Proteins

The strains of influenza A viruses used for the NP expression (Table 1) were obtained from the influenza virus repository of the Institute of Experimental Medicine or purchased from the collection of Smorodintsev Research Institute of Influenza (St. Petersburg, Russia).

The full-length recombinant 6xHis-tagged NPs of ten evolutionary distant influenza A strains of different subtypes were expressed in *E. coli* BL21(DE3) cells by 0.5 mM IPTG (Sigma, St. Louis, MO, USA) induction and further purified by immobilized metal affinity chromatography similar to the previously described approach [14]. The obtained antigens were identified by mass spectrometry and serological methods as described below.

### 2.2. SDS-PAGE and Western Blot

Sodium dodecyl sulfate–polyacrylamide gel electrophoresis (SDS-PAGE) [15] was used to check the structure and purity of obtained NPs, while the ability of anti-NP mAbs to detect linear epitopes of NP proteins of ten influenza strains was assessed via Western blotting. Purified recombinant NP proteins were resolved in reduced conditions on a 10% polyacrylamide gel at 120 V for 1 h before being stained with colloidal Coomassie G-250 solution (Bio-Rad, Hercules, CA, USA) for 1 h at room temperature or semi-dry transferred to 0.45 μm nitrocellulose membranes for 2 h at 100 V. Blots were blocked overnight at 4 °C with 5% skimmed milk in PBS-T and then treated with anti-NP mAbs 6D11 diluted 1:1000 in block buffer for 1 h at 37 °C. Then, goat anti-mouse HRP-conjugated secondary antibody (Bio-Rad, Hercules, CA, USA) diluted 1:3000 in blocking solution was added to the triple-washed blots for 1 h at 37 °C. After three washes with PBS-T, the blots were developed with 0.05% solution of diaminobenzidine (Sigma, St. Louis, MO, USA) in PBS containing 1% hydrogen peroxide. Finally, the membranes were washed with water and the images were captured using Gel Doc EZ Gel Documentation System (Bio-Rad, Hercules, CA, USA).

### 2.3. MALDI-TOF Mass Spectrometry

Mass spectrometric identification of proteins was performed according to [16]. To analyze the amino acid sequence of proteins after SDS-PAGE, enzymatic hydrolysis in gel by trypsin was performed. Fragments of the stained areas were excised (~1 mm^3^), washed from the dye (twice 150 μL each of 30 mM ammonium bicarbonate solution, 40% acetonitrile in water), and dehydrated in 100% acetonitrile, after which acetonitrile was withdrawn and the gel fragments were incubated in air until complete evaporation of acetonitrile. Then, 2 μL of trypsin solution (20 μg/mL in 50 mM ammonium bicarbonate) was added to the gel fragments and incubated at 37 °C for 18 h. The reaction was stopped with 3 μL of a solution of 1% THF, 10% acetonitrile in water. For protein identification, the resulting set of tryptic peptides were mixed with DHB matrix (Bruker, Billerica, MA, USA) in equal volumes, applied to a steel target, and examined in reflectance positive ion detection mode on an UltrafleXtreme MALDI-TOF/TOF mass spectrometer (Bruker, Billerica, MA, USA). At least 5000 laser pulses were summed for each spectrum. Protein identification was performed using MASCOT search engine [17] by simultaneously accessing the UniProt database [18]. Methionine oxidation and deamidation were indicated as variable modifications. The accuracy of mass determination was limited to 20 ppm. Up to two trypsin errors (omission of a proteolysis site) were allowed.

### 2.4. Immunizations

In order to study the effect of adaptive NP mutations on its antigenic and immunogenic properties, rNPs served as antigens for triple mice immunization. BALB/c mice (females, 16–18 g, 6–8 weeks old, 10 animals per group, which was the minimum required for proper statistical assessment; one hundred mice in total) were purchased from Rappolovo cattery (Leningrad region, Russia). The experimental design was approved by the local ethics committee of the Institute of Experimental Medicine (protocol no 4/24, dated 24 October 2024). The animals were maintained in standard laboratory vivarium conditions with free access to food and water. The study was conducted in accordance with Directive 2010/63/EU [19]. The experiment was designed to use the smallest possible number of animals that still met statistical requirements, following the “Three Rs” principles.

Animals were pre-screened in ELISA on 2 µg/mL immobilized rNP of A/Leningrad/134/17/57 (H2N2). In order to study the effect of adaptive NP mutations on its antigenic and immunogenic properties, rNPs served as antigens for triple BALB/c mice immunization (intraperitoneally, 15 µg/animal at 14-day interval). The antigens were adjuvanted with aluminum hydroxide (1:3 *v*/*v*). The immunizations were performed in strict order, the animals were marked with 0.5% picric acid, and cages were placed in different locations to minimize potential confounders. All immunizations were performed by trained personnel who were aware of the group allocation; however, all subsequent serum analyses were performed blinded. Total blood collection was performed on the 42nd day from the first immunization humanely under mild ether anesthesia. The sera were isolated by the centrifugation at 3500 rpm for 15 min, aliquoted and stored at −20 °C.

### 2.5. Assessment of NP-Specific Humoral Responses

Serum NP-specific antibody levels were measured by solid-phase ELISA. For this, high-binding 96-well plates (Thermo Fisher Scientific, Waltham, MA, USA) were coated with previously obtained rNPs, 100 ng per well, in a carbonate–bicarbonate buffer (pH 9.5) overnight at 4 °C. Then, the blocking was performed with 1% BSA in PBS (pH 7.4) for 40 min at 37 °C followed by the triple washing with PBS-T (PBS with 0.1% Tween 20). Serum sample dilutions included one 10-fold and several 5-fold in PBS-T (1:100 and 1:500 to 1:121,500), which, in duplicates, was then incubated in wells for 1 h at 37 °C. As a detecting secondary antibody, goat anti-mouse IgG HRP conjugate (Bio-Rad, Hercules, CA, USA) was added at the next step to the washed plates for 1 h at 37 °C. After the final triple washing, the plates were developed with 1-Step TMB Substrate Solution (HEMA, Moscow, Russia) for 15 min. The reaction was stopped with 1 M of H_2_SO_4_, and the resulting optical densities (OD_450_) were measured at a wavelength of 450 nm using an xMark Microplate Spectrophotometer (Bio-Rad, Hercules, CA, USA). The serum titers have been determined as final dilutions at which OD_450_ values exceeded twice those of the negative controls. The area under the OD_450_ curve (AUC) values were then calculated by the integration for each serum sample and expressed in arbitrary units.

### 2.6. Statistical Analysis

Statistical data processing was performed in GraphPad Prism 6.0 Software. The Shapiro–Wilk’s test was used to check the compliance with normal distribution. The Wilcoxon matched-pairs test was used to compare serum humoral responses to different rNPs. Data were compared using one-way ANOVA with Tukey’s post hoc test. A significance level was set at *p* < 0.05.

## 3. Results

### 3.1. Expression and Identification of Recombinant NPs

**Figure 1:**
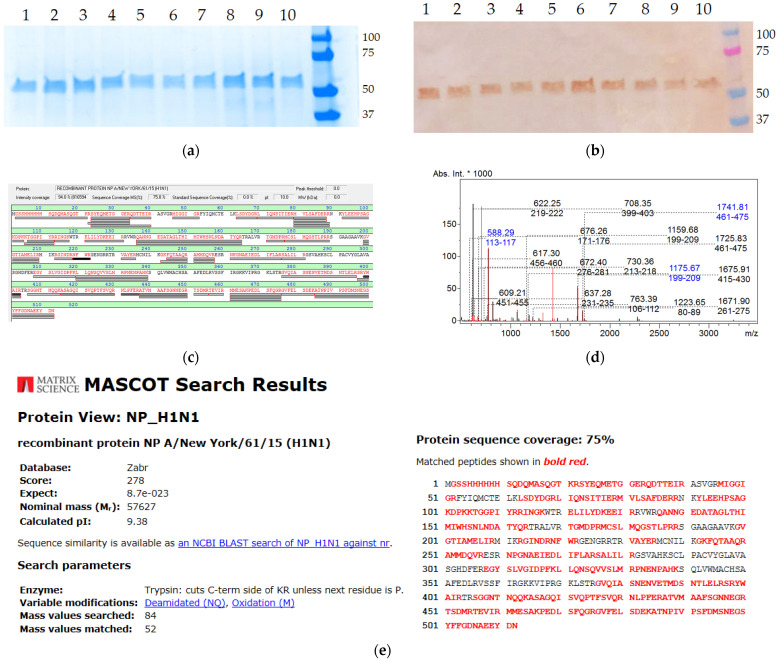
SDS-PAGE (**a**) and Western blot (**b**) analyses of the purified recombinant NP antigens and example of mass spectrometry identification: sequence coverage (**c**), peptide spectrum (**d**), matching with database sequence (**e**). The SDS-PAGE was performed in reducing conditions (in the presence of 1% β-mercaptoethanol).

The purified recombinant NP antigens were expressed in *E. coli* BL21 (DE3) system and further characterized by SDS-PAGE (Figure 1a) and Western blot (Figure 1b) assays, as well as being identified by MALDI-TOF analysis (Figure 1c,d). The electrophoretic mobility of the antigens corresponded to the expected molecular weights (~55 kDa in reducing conditions). The SDS-PAGE analysis of the purified NPs demonstrated the absence of significant amounts of contaminating components. All the recombinant proteins were detected by the anti-NP monoclonal antibodies 6D11 [20] and uniquely identified by mass spectrometry, since the detection score was well above the threshold value of 70 (Figure 1e).

### 3.2. Different rNPs Exhibit Distinct Humoral Immunogenicity in Mice

**Figure 2:**
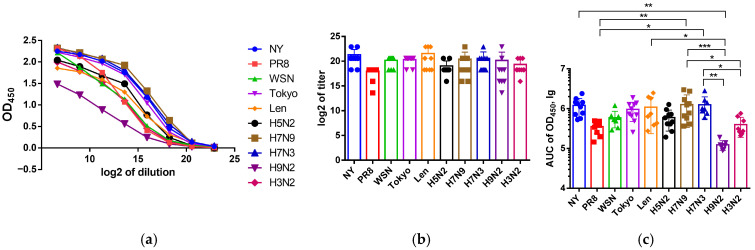
Assessment of homologous anti-NP antibody levels in serum samples of mice immunized with different rNPs. (**a**) OD_450_ values at different serum dilutions. (**b**) Endpoint titers of serum IgG antibodies. (**c**) The area under the OD_450_ curve (AUC) values are shown. The data were compared by ANOVA with Tukey’s post hoc test. * *p* < 0.05, ** *p* < 0.01, *** *p* < 0.001.

The magnitudes of IgG responses elicited in mice immunized with NP proteins of master donors were also defined as OD450 values, which are given as titration curves in Figure 2. In general, the intensity of antibody binding to the heterologous NP antigens was slightly reduced compared to that of the homologous protein, and the overall antibody response to NP H3N2 was lower than that to NP H1N1 or H2N2. The intensities of IgG generation in mice in response to immunization with “avian” NP proteins are also shown as OD450 values in Figure 2. Despite the general similarity of humoral responses of different sera groups to four NPs, a common decrease in affinity for NP H9N2 was revealed.

As can be seen from Figure 2, the AUC calculation turned out to be more informative compared to the titer determination, especially for the anti-H9N2 antibodies, so we subsequently used this strategy to analyze the sera cross-reactivity.

### 3.3. Cross-Reactivity of Anti-NP Antibodies Induced in Mice by rNPs

Pronounced cross-reactivity of anti-NP antibodies induced in mice by immunization with different rNPs was observed. At the same time, we observed the differences in rNP immunogenicity as the ability to induce different levels of anti-NP antibodies, which are in line with the assumptions on slow accumulation of evolution-driven NP mutations. In general, antibody affinity to heterologous rNPs was reduced, indicating the differences in the specificity of anti-NP immunoglobulins, which may be caused by evolutionarily determined variability of immunogenic B-cell epitopes leading to the emergence of escape mutations.

**Figure 3:**
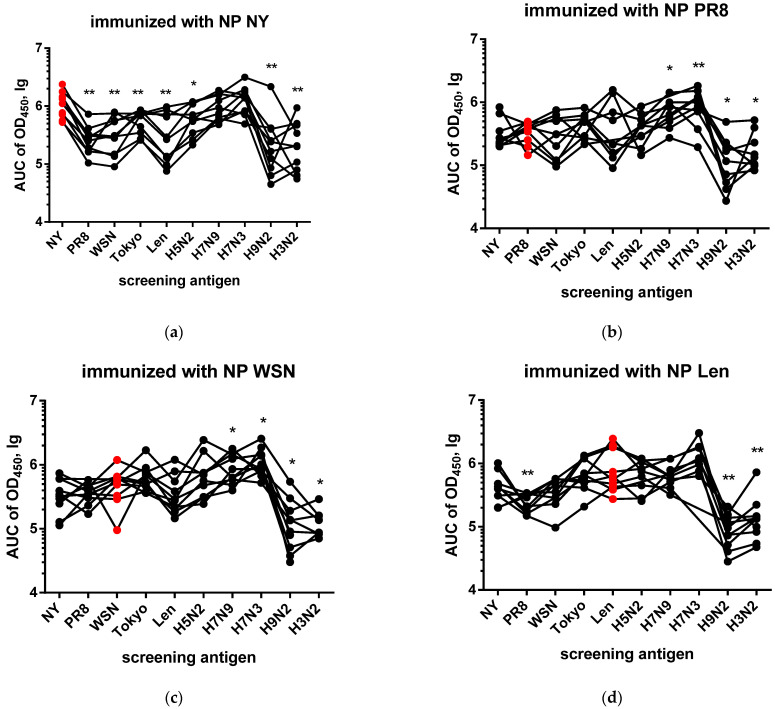

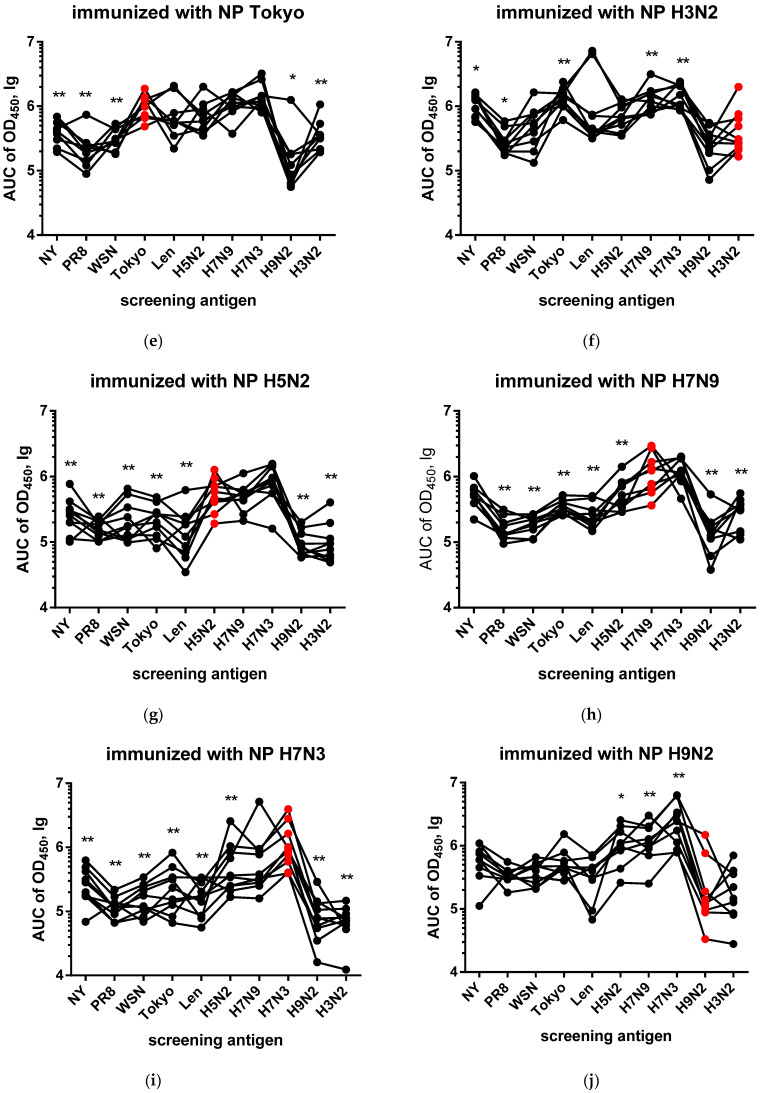
NP-based ELISAs of serum samples of mice immunized with NP NY (**a**), NP PR8 (**b**), NP WSN (**c**), NP Len (**d**), NP Tokyo (**e**), NP H3N2 (**f**), NP H5N2 (**g**), NP H7N9 (**h**), NP H7N3 (**i**), NP H9N2 (**j**). The red circles indicate the animals immunized with the homologous antigen, The data were compared using the Wilcoxon’s matched-pairs test. The logarithmic OD_450_ values are shown. *—*p* < 0.05, **—*p* < 0.01.

As expected, analysis of sera from mice immunized with different NPs revealed significant cross-reactivity of antibodies to NP (Figure 3), which indicates the presence of highly conserved B-cell epitopes in its structure. The immunization of mice with NP NY (Figure 3a) induced a high level of antibody response to the homologous antigen. When compared with heterologous NP PR8, NP WSN, NP Tokyo, NP Len, NP H5N2, NP H9N2 and NP H3N2, statistically significant differences were observed (* *p* < 0.05; ** *p* < 0.01). In response to immunization with NP PR8 (Figure 3b), antibodies with altered specificity to NP H7N3, H7N9, NP H9N2 and NP H3N2 were generated (* *p* < 0.05; ** *p* < 0.01), while no differences in specificity were found on NP NY, NP WSN, NP Len, NP Tokyo and NP H5N2. Similarly, immunization with NP WSN (Figure 3c) induced a pronounced humoral response against NP of H1N1, H2N2 and H5N2 subtypes, while statistically significant differences were also observed for NP of H7N9, H7N3 H9N2 and H3N2. The NP Len immunized group (Figure 3d) demonstrated high AUC450 values to the homologous antigen. In this case, significant differences were revealed when comparing the response to the homologous antigen with those against NP PR8, NP H9N2 and NP H3N2 (* *p* < 0.05; ** *p* < 0.01). Immunization with NP Tokyo (Figure 3e) also resulted in a strong homologous immune response, and significant differences were detected upon the screening on NP of H1N1, H9N2 and H3N2 subtypes (* *p* < 0.05; ** *p* < 0.01). In the group immunized with NP H3N2 (Figure 3f), the highest AUC450 values were detected for the homologous antigen, and significant differences in antibody reactivity were revealed on NP NY, NP PR8, NP Tokyo, NP H7N9 and NP H7N3 (* *p* < 0.05; ** *p* < 0.01).

Screening on immobilized NP H5N2 (Figure 3g) revealed a high level of cross-reactivity in all groups, which did not differ statistically. The only exception was a difference between the NP PR8 and NP H9N2 groups (* *p* < 0.05). When screened on the immobilized NP H7N9 (Figure 3h), sera from the NP H3N2 group was significantly different in properties from the NP H7N3 and NP H5N2 groups (* *p* < 0.05). In the case of the NP H7N3 antigen (Figure 3i), the absence of statistically significant differences in sera reactivity was noted. On NP H9N2 (Figure 3j), all sera demonstrated a high level of binding, with sera against NP H3N2 being statistically more reactive compared to the NP WSN, NP Tokyo, NP Len, NP H5N2, NP H7N3, and NP H9N2 groups (* *p* < 0.05, ** *p* < 0.01).

Thus, despite the high conservancy, different NPs possess the feature of generating different subsets of anti-NP antibodies. Our data from the NP (pdm09) and NP (H7N3′00) immunization groups suggest that NP-specific antibodies raised to infection with evolutionarily new strains will differ from those produced in response to “old” rNP administration.

Thus, the highest values of AUC OD450 were detected for the NP H7N9, NP WSN, NP NY and NP H7N3 groups, which formed a cluster with the most pronounced humoral immunogenicity. The NP Len and NP Tokyo groups demonstrated intermediate values. Minimal values were observed for NP H3N2, NP PR8 and NP H9N2.

**Figure 4:**
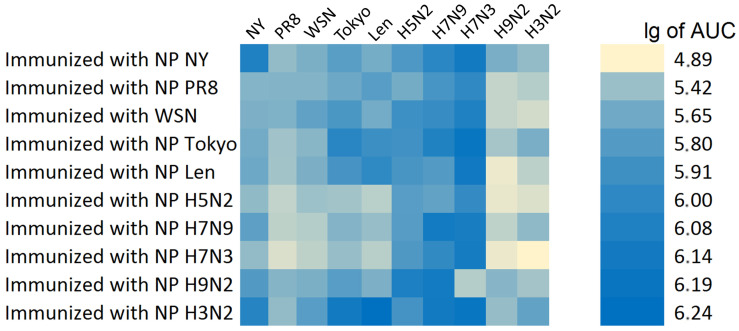
Heat map of interactions of anti-NP sera with homo- and heterologous recombinant antigens. The area under the OD_450_ curve (AUC) values are shown.

The summary results of the determination of the cross-specificity of anti-NP antibodies in relation to various recombinant antigens are presented in Figure 4. The strongest reactivity was observed for NP immunogen of subtypes H7N9, H7N3, H2N2 and H1N1pdm09.

### 3.4. Differences in Amino Acid Composition of Studied rNPs

**Figure 5:**
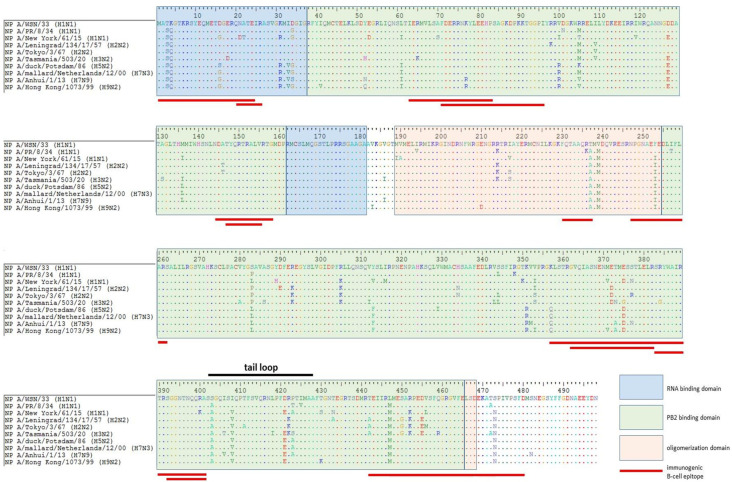
Multiple alignment of NP sequences for A/WSN/1933 (H1N1) influenza virus, H1N1pdm09 and bird influenza strains supplemented with the domain NP structure and localization of B-cell immunogenic epitopes. Different NPs of the virus are shown. Amino acid positions in the NP protein (from 1 to ~498) are indicated with the colored text, for which amino acid differences are displayed (substitutions are shown as amino acid letters, matches are shown as dots); color coding corresponds to the functional domains of NP (blue—RNA-binding domain, green—PB2-binding domain, pink—oligomerization domain). Red horizontal lines indicate the location of immunogenic B-cell epitopes. In the amino acid sequence region 402–428, the tail loop element involved in oligomerization is marked. The figure was generated using GISAID data and BioEdit v7.2.5 [21].

As shown in Figure 5, a number of mutations had occurred in the NP sequences of more recent viruses (mostly in RNA-/PB2-binding and tail loop domains), suggesting that B cells targeted to some NP epitopes of the developed NP-based vaccine candidates, as well as to those of the licensed IIVs/LAIVs, may not recognize the corresponding proteins of currently circulating influenza A viruses.

A significant number of adaptive substitutions are localized within experimentally detected immunogenic B-cell epitopes, and it is very unlikely that antibodies produced in response to vaccination with the non-mutated NP will recognize “new” epitopes containing mismatched residues.

As alignment analysis shown, the relative frequency of amino acid substitutions in different functional domains ranges from 14 to 18% (RNA-binding domain ~57 amino acid residues and 9 substitutions, ~15%; PB2-binding domain ~336 amino acid residues and 63 substitutions, ~18%; oligomerization domain ~69 amino acid residues and 10 substitutions, ~14%).

**Figure 6:**
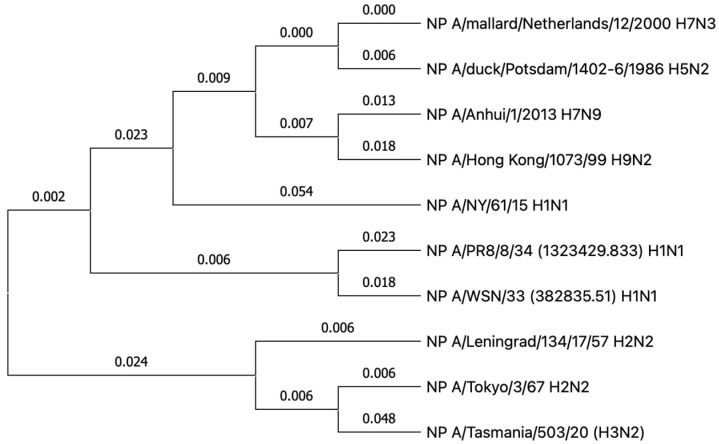
Phylogenetic relationship of studied NPs of different influenza A virus subtypes. The expected number of substitutions per site (nucleotide) between two sequences since their divergence is shown. The data analysis and visualization was performed in MEGA 11 Software (Test Maximum Likelihood Tree, Bootstrap method—1000, Model/Method: JTT, Rates Among Sites: Gamma Distributed (G), Use all sites) [22].

The observed differences in the cross-reactivity of the anti-NP antibody may be partially explained by the evolutionary relationships of these antigens in different influenza A virus strains (Figure 6).

The phylogenetic tree of the NP of influenza A viruses demonstrates evolutionary divergence between NP NY H1N1 and all other NPs analyzed. The remaining protein sequences form two clades. The first is made up of the NP of influenza virus of avian origin, within which the pairs NP H7N3 and NP H5N2, as well as NP H7N9 and NP H9N2, are evolutionarily close. The second group is made up of the NP of the influenza viruses that are pathogenic for humans, where a close relationship is observed between NP H1N1 (NP PR8 and NP WSN), which, in turn, are united by a common origin with the cluster containing NP Leningrad H2N2, NP Tokyo H2N2 and NP H3N2. At the same time, the NP H3N2 branch demonstrates an accumulation of a greater number of amino acid substitutions, in comparison with NP Tokyo H2N2. Thus, the tree illustrates the evolutionary distance between NP NY H1N1 and the two major clades of influenza A viruses that are able to propagate in birds and humans.

## 4. Discussion

The question of whether NP should be updated in modern whole-virion influenza vaccines remains open [23]. The high degree of NP conservation makes it an attractive target for the development of universal vaccines, diagnostic systems [3] and even engineered biosensors [24]. However, some studies note that, despite the evolutional preservation of the NP epitopes, its antigenic features still differ significantly between the strains and subtypes of the influenza virus [25]. The differences in the immune response to the NP of circulating influenza strains and older variants underlie the results obtained, indicating that changes in immunodominant NP regions led to the limited effectiveness of existing vaccines based on “outdated” NP variants.

In this study, the cross-reactivity of anti-NP antibodies as well as the humoral immunogenicity of different NPs were investigated. It is important to note that the present study did not assess overall NP immunogenicity (which could also involve T-cell responses) but rather focused only on differences in anti-NP antibody responses. Cross-reactivity analysis of antibodies induced in mice by immunizations with NP of vaccine donors (Figure 3) revealed the presence of pronounced cross-specificity. The antibodies demonstrated the greatest reactivity to homologous NPs, but retained the ability to bind to proteins of other subtypes. A wide range of heterologous sera bind to NP H3N2 and NP Len (Figure 3d,f) at a high level. These results may indicate that these NPs have retained the greatest number of conserved epitopes common to many subtypes of the influenza A virus. Similar patterns are observed in cross-reactivity assay on immobilized avian NPs (Figure 4 and Figure 5). The signal intensities observed on NP H5N2 and NP H7N3 (Figure 3g,i) were relatively uniform, which reflects the presence of common immunogenic NP epitopes conserved in the avian subtypes. This also correlates with their evolutionary close relationship reflected in the phylogenetic tree (Figure 6). The tendency towards a decrease in cross-reactivity was more pronounced for the NP H9N2 immunogen (Figure 3 and Figure 4). This is probably due to the low similarity of its epitopes to the proteins of human subtypes, which is confirmed by its phylogenetic position in the avian clade. The only exception is NP H3N2, the unusually high reactivity of antibodies, which may indicate the similarity of individual epitopes with NP H9N2 (Figure 3f). Analysis of homologous humoral NP responses (Figure 2) showed that NP H7N9, NP WSN, NP NY and NP H7N3 were the most reactive, while NP H9N2, NP PR8 and NP H3N2 caused a weak humoral response. This indicates that the conservative NP is also subjected to slow evolutionary changes that can reduce the effectiveness of the induced response. The alignment of NP sequences of different influenza A viruses (Figure 5) showed that the greatest evolutional variability is concentrated in the PB2-binding domain (~18%). Most of the substitutions are located within the B-cell epitopes, which reflects the mechanism of influenza virus evolution—a strategy to evade the host’s immune response.

The obtained results indicate that the most pronounced immune responses were detected in relation to the homologous antigen. We found that immunization with NP of some influenza subtypes induced a cross-specific response against a limited number of other subtypes. This confirms the previously obtained data on the conservatism and broad, cross-reactive immune response against NP [26]. However, in some cases, the affinity of antibodies to heterologous antigen was comparable or even exceeds the binding intensity to the homologous one (Figure 3). This may be attributable to specific amino acid substitutions that enhance antibody affinity (Figure 5), but these results require additional studies. This phenomenon has been previously described for other viral antigens, where heterologous antibodies showed a stronger reactivity with some distant protein variants than homologous ones [27]. In cases of screening on avian NPs, homologous responses were also strong, but differences in reactivity to the closest avian NPs were often statistically insignificant or absent, which may indicate a low rate of evolution among NPs of avian influenza strains [28]. This is consistent with the results presented in previous reports stating that avian NPs do not have significant evolutionary proximity to NPs of influenza viruses pathogenic for humans [29]. By comparing the NPs of vaccine donors and avian NPs, the characteristic differences in the cross-reactivity profiles were identified. While in the first case the cross-reactivity was expressed variably between different NP pairs, the avian NPs differ in their mutual cross-reactivity. This is in line with a study where antibodies to the NP of avian influenza viruses react equally well with different avian subtypes, since their NPs are highly conserved. At the same time, the NP sequences of mammalian influenza viruses differed more from each other, so the cross-reactivity between them was variable [30]. These facts create an overall picture of the consistency of NP evolution with anti-NP antibody reactivity profiles. Our results challenge the conventional view of NP as a strictly conserved protein and demonstrate that even slow evolutionary changes may affect the antigenic properties of this intrinsic viral protein.

Based on the data obtained, it can be concluded that the influenza virus nucleoprotein exhibits a dual nature: on the one hand, it is rather conservative and antigenic [29], and on the other hand, it is capable of changing in certain immunodominant epitopes, which represents a key immune evasion mechanism [31].

Obviously, our study has several limitations. First, the experimental groups were relatively small and the weights of the animals were not absolutely identical, which could increase the variability of the data. Second, a large dilution factor and a limited number of dilutions were used for ELISA, which ensured the possibility of AUC calculation and plotting the titration curve, but limited its smoothness and the accuracy of titer assessment. Moreover, some of the serum antibodies were apparently raised against the 6xHis tag contained in the recombinant NPs, which could have had a minor effect on the cross-reactivity analysis results. Next, NP was administered intraperitoneally, whereas during natural infection the antigen is introduced intranasally. This allowed us to obtain hyperimmune sera, but did not reproduce the natural conditions of infection. In addition, the study used recombinant antigens rather than viral strains, although mass spectrometric and immunochemical identification was performed. Finally, only the humoral response was assessed, whereas NP, as an internal protein of the virion, is considered to be an effective inducer of T-cell immunity [4].

Here, other key immunological studies were not performed (T-cell responses, viral challenges, etc.), but the obtained results on the humoral immunogenicity indirectly characterize the immunomodulating NP ability and thus support its potential as a target for the development of tools to combat influenza infection.

In the future, it would be advisable to expand our research. For example, to analyze the T-cell response to various NPs, the number of sera dilutions should be increased (as the used dilution scheme with one 10-fold and several 5-fold steps may have affected the quantitative intergroup comparisons), as should the number of experimental animals to improve statistical reliability, and the immunogenicity of recombinant NPs and NPs produced during natural infection should be compared. Immunization with intranasal administration of the virus will allow a more accurate assessment of the contribution of adaptive mutations of NP to changes in its antigenic properties, and will provide grounds for discussing the need to update NP in the composition of live attenuated and inactivated influenza vaccines.

## 5. Conclusions

Overall, our results reflect the slowly evolving nature of the NP antigen, which influences the specificity spectrum of anti-NP antibodies and should be recognized as a limitation for the development of NP-based cross-protective vaccines and test systems. In particular, the data obtained indicate that NP-specific antibodies generated in response to immunization with antigens of classical donors are capable of recognizing only a part of the epitopes of the NP antigen of modern influenza virus variants, which may have at least two negative consequences. First, the mass use of vaccines based on irrelevant NP donors will lead to clonal expansion of effector B lymphocytes incapable of producing antibodies against NPs of recent influenza virus variants, which will unnecessarily deplete the immune system of vaccinated individuals. Second, most of the new NP epitopes of modern influenza viruses will not be recognized by vaccine-induced B cells, since these epitopes have not yet been presented in vaccines based on classical donors.

This drawback can be eliminated either by introducing into the composition of whole-virion vaccine strains, in addition to the genes of surface proteins (hemagglutinin and neuraminidase), the NP gene of current viral variants, or by targeted point mutagenesis of the NP sequences of vaccine donors to bring it into line with the antigenic set of B-cell NP epitopes of modern influenza A viruses.

Thus, there is a possibility of limited cross-NP specificity, and, consequently, the protective function of vaccine-induced anti-NP antibodies, which can be corrected by updating the epitope composition of NP vaccine donors in accordance with that of current viral variants to obtain new prototypes of influenza vaccines with improved cross-protective properties.

## Figures and Tables

**Table 1:** The strains used for NP expression in the article.

Subtype	Strain	Abbreviation in the Article
H1N1	A/WSN/33	WSN
H1N1	A/PR/8/34	PR8
H1N1pdm09	A/New York/61/15	NY
H2N2	A/Leningrad/134/17/57	Len
H2N2	A/Tokyo/3/67	Tokyo
H3N2	A/Tasmania/503/20	H3N2
H5N2	A/duck/Potsdam/86	H5N2
H7N3	A/mallard/Netherlands/12/00	H7N3
H7N9	A/Anhui/1/13	H7N9
H9N2	A/Hong Kong/1073/99	H9N2

## Data Availability

The raw data that support the findings of this study are publicly available on the Yandex Disk Repository at: [https://disk.360.yandex.ru/d/Wv9S8w6aAgOpAA] (accessed on 11 May 2026).
